# Fe^3+^-Sensitive Carbon Dots for Detection of Fe^3+^ in Aqueous Solution and Intracellular Imaging of Fe^3+^ Inside Fungal Cells

**DOI:** 10.3389/fchem.2019.00911

**Published:** 2020-01-15

**Authors:** Yanqiu Chen, Xiaobo Sun, Wei Pan, Guifeng Yu, Jinping Wang

**Affiliations:** College of Chemical and Pharmaceutical Sciences, Qingdao Agricultural University, Qingdao, China

**Keywords:** carbon dots, Fe^3+^, fungal cells, bioimaging, detection

## Abstract

In this article, the Fe^3+^-sensitive carbon dots were obtained by means of a microwave-assisted method using glutamic acid and ethylenediamine as reactants. The carbon dots exhibited selective response to Fe^3+^ ions in aqueous solution with a turn-off mode, and a good linear relationship was found between (F_0_-F)/F_0_ and the concentration of Fe^3+^ in the range of 8–80 μM. As a result, the as-synthesized carbon dots can be developed as a fluorescent chemosensor for Fe^3+^ in aqueous solution. Moreover, the carbon dots can be applied as a fluorescent agent for fungal bioimaging since the fungal cells stained by the carbon dots were brightly illuminated on a confocal microscopy excited at 405 nm. Furthermore, an increase in the concentration of intracellular Fe^3+^ could result in fluorescence quenching of the carbon dots in the fungal cells when incubated in the Tris-HCl buffer solution containing Fe^3+^. However, due to EDTA might hinder Fe(III) to enter the fungal cells, incubation in Fe(III)-EDTA complex solution exerted negligible effect on the fluorescence of fungal cells labeled by the carbon dots. Therefore, the carbon dots can serve as a potential probe for intracellular imaging of Fe^3+^ inside fungal cells.

## Introduction

As the most abundant transition metal in cellular systems, iron with its chemical versatility plays indispensable roles in physiological processes (Gao R. et al., 2019) such as oxygen transport (Aisen et al., 1999), electron transfer (Lucas et al., 2011), and enzymatic catalysis (Eisenstein, 2000). Excess or deficiency will cause damages to live systems (Bischof et al., 2019). Therefore, it is of great significance to detect iron ions with high sensitivity and selectivity in both environmental and biological systems.

Fluorescent sensors have attracted increasing attention for the detection of metal ions, owing to their simplicity, high selectivity and sensitivity, high spatial resolution, and possibility for real-time, rapid monitoring (Dean and Palmer, 2014; Huang et al., 2014; Li and Liu, 2014). Moreover, metal ions monitoring in cells and organisms can be visually achieved by imaging based on fluorescent sensors. Generally, most widely used fluorescent sensors include fluorescent metal nanoclusters (Shang et al., 2011), organic dyes (Wang et al., 2019), semiconductor quantum dots (QD) (Wu et al., 2003), fluorescent metal organic frameworks (Ma et al., 2014). But concerns may come from their photo instability, complex equipment and treatment processes, high costs, environmental unfriendliness, and cytotoxicity in their practical applications.

Recently, carbon dots have interested the research community as bioimaging labels and sensors due to their high performances, including bright fluorescence (Zhu et al., 2013), excellent photostability (Zhang et al., 2019), tunable fluorescence emission (Sun et al., 2006), favorable biocompatibility (Deng et al., 2019), low toxicity (Ye et al., 2019), and good water solubility (Gao Y. et al., 2019). As presented in the available documents, most of carbon dots showed great potential in bioimaging of different types of cells. For instance, imaging of bacterial cells, including *Escherichia coli*, and *Mycobacterium tuberculosis, Pseudomonas aeruginosa*, even visualization of physiological processes such as cell division, were achieved using carbon dots as fluorescent label (Mehta et al., 2015; Nandi et al., 2015; Shi D. et al., 2015). Lots of carbon dots synthesized by various carbon sources and surface modified by different soft molecules showed bright fluorescence, and have been successfully applied in imaging of various human cells with low cytotoxicity, such as A193, SCC-7, A549, Hep-2, and MCF-7 cells (Shi L. et al., 2015; Wang et al., 2015; Zheng et al., 2015; Zhu et al., 2015; Zhuo et al., 2015). On the other hand, a variety of methods based on carbon dots with high sensitivity and selectivity have been reported for chemically sensing Hg^2+^ (Yan et al., 2016), Cu^2+^ (Zhu et al., 2017), Fe^3+^ (Tian et al., 2017), Pb^2+^ (Xiong et al., 2016), Cr(VI) (Wang et al., 2017), and Ag^+^ (Jiang et al., 2015). Furthermore, it is of great interest for intracellular imaging, and even quantitatively detecting specific metal ions in cells with high sensitivity and selectivity using carbon dots as fluorescent probes. For example, the nitrogen and phosphorus doped carbon dots had been reported for the detection of Fe^3+^ ions in cancer cells (Teng et al., 2014). And a dual-emission nanosensor based on carbon dots exhibited high performance for the detection and ratiometric fluorescence imaging of Cu^2+^ in cells (Qu et al., 2012).

Iron also plays an important role in physiological activities of fungal cells. To image and detect intracellular Fe(III), the physiological and pathological processes of the cells will be better understood. Cell imaging and visualizing intracellular pH variation of fungi had been achieved by our group using carbon dots as a fluorescent probe prepared by facile one-pot hydrothermal pyrolysis of threonine (Zhou et al., 2015). However, to our knowledge, no reports were found to apply carbon dots as probes in the bioimaging of intracellular Fe^3+^ inside living fungal cells. In this article, the microwave-assisted method were applied to prepare Fe^3+^ sensitive carbon dots using glutamic acid and ethylenediamine as reactants without any further modification. The as-synthesized carbon dots will be a potential candidate for optical detection of Fe^3+^ in aqueous solution, bioimaging of fungal cells, and intracellular imaging of Fe^3+^ inside fungal cells.

## Experimental

### Synthesis of the Carbon Dots

Materials, characterization methods and cytotoxicity to the fungus of the carbon dots are shown in the Supplementary Material.

The carbon dots were prepared by microwave assisted pyrolysis of L - glutamic acid and anhydrous ethylenediamine as raw materials (Lu et al., 2015; Li et al., 2016). Briefly, 500 mg of L - glutamic acid was dissolved in 20 mL ultrapure water to get homogeneous solution, into which 1.65 mL anhydrous ethylenediamine was slowly added. Subsequently, the resulting mixture was heated by a domestic microwave oven for 4 min. Then, another 10 mL of ultrapure water was added to dissolve the product after it naturally cooled down to room temperature. Thereafter, the obtained solution purified by dialyzing against ultrapure water through a dialysis membrane (MWCO 500) for 2 days. The purified solution was concentrated by means of rotary evaporation at 50 °C. Finally, the solid carbon dots were obtained by freeze drying under vacuum at ambient temperature for 48 h.

### Detection of Metal Ions

Typically, 0.15 mg/mL of the carbon dots were mixed with Fe^3+^ at various concentrations in Tris-HCl buffer (0.1 M, pH 7.0) solution, then their fluorescence spectra were collected at room temperature. The relative fluorescence intensity (F_0_-F)/F_0_ was calculated to be the signal output value, in which F_0_ and F represent the fluorescence intensities at 460 nm of the carbon dots in the absence and presence of Fe^3+^, respectively.

### Detection of Fe^3+^ in Real Samples

To evaluate the feasibility of the carbon dots based sensor for the detection of Fe^3+^ in real samples, tap water samples obtained from our lab were analyzed using the present method. All water samples were spiked with Fe^3+^ at different concentrations without any pretreatment.

### Imaging of Fungal Cells

Fungal fermentation was carried out in liquid medium at 27 ± 2 °C for 3 days. Thereafter, the selected young vigorous mycelia washed by distilled water for three times were incubated in the Tris-HCl buffer solution containing the as-synthesized carbon dots at a certain concentration for another 36 h in a constant temperature incubator. The stained mycelia were imaged on a laser scanning confocal microscopy (Lieca, Germany) after washed three times with distilled water. Three drops of 0.005 M Fe^3+^, Fe(III)-EDTA, Cu^2+^, and Ca^2+^ were added from one side of the chink between the cover glass and the slide glass for intracellular imaging of metal ions.

## Results and Discussion

### Characterization

**Figure 1:**
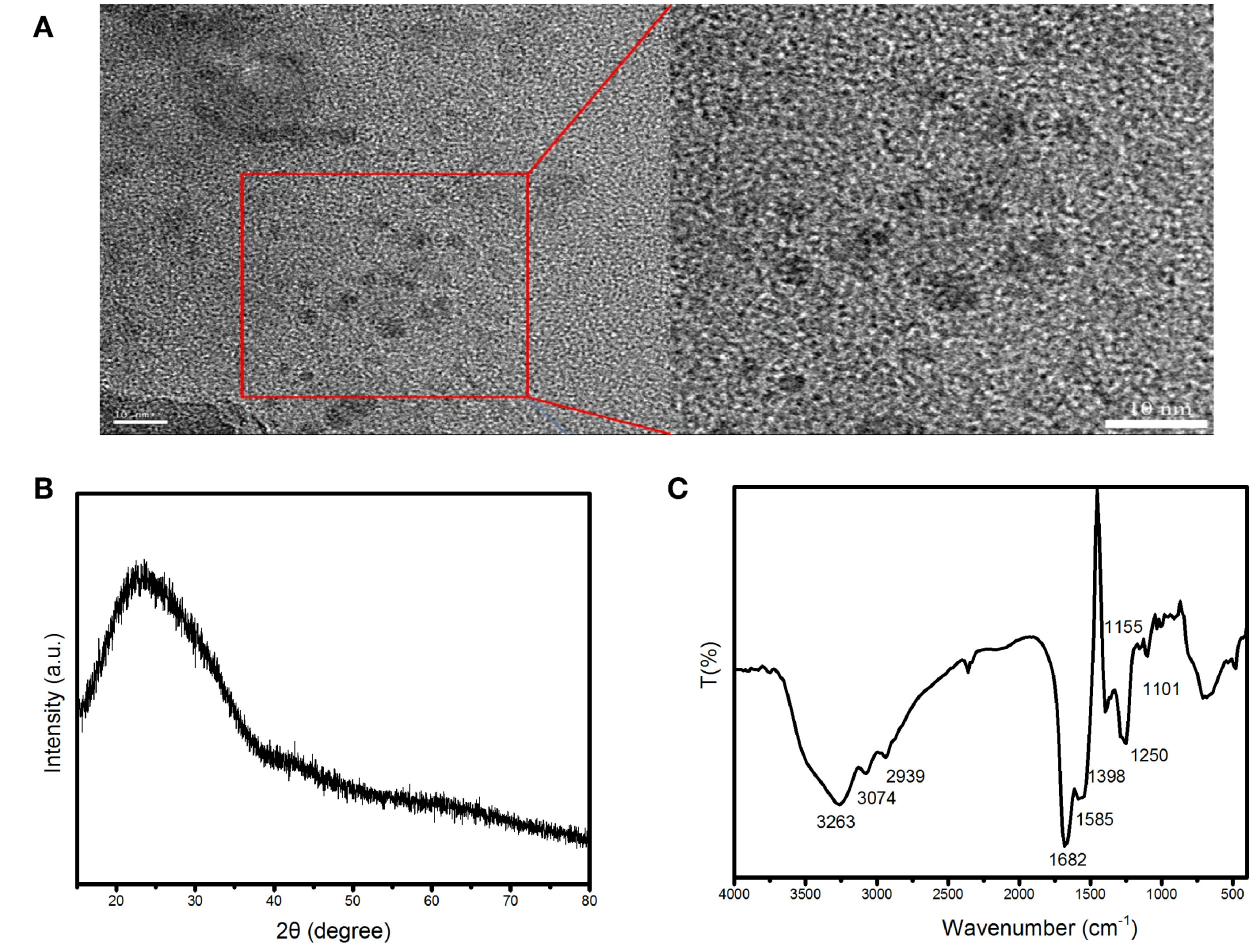
**(A)** TEM image, **(B)** XRD pattern, and **(C)** FTIR spectrum of the as-synthesized carbon dots.

TEM image of the as-synthesized carbon dots is shown in Figure 1A. The morphology of the carbon dots is nearly spherical, and their average diameter is 4.5 nm. The measured XRD pattern of the carbon dots showed an obvious peak centered at 2θ = 23.3° with *d* = 0.381 nm (Figure 1B), which is corresponding to the (002) lattice spacing of carbon based materials (Vikneswaran et al., 2014). And the functional groups on the surface of the carbon dots were identified by FTIR. As demonstrated in Figure 1C, the broad band centered at 3,263 cm^−1^ was contributed by the stretching vibrations of –OH and –NH_2_. The absorption at 3,074 cm^−1^ was attributed to the stretching vibration of C=C (Dong et al., 2012). The peaks at 1,682, 1,585, 1,250, and 1,101 cm^−1^ belonged to C=O stretching vibration, N-H bending vibration, C-OH stretching vibration and C-N stretching vibration. The FTIR information suggests that there are carboxyl and amine groups on the surface of the as-synthesized carbon dots.

**Figure 2:**
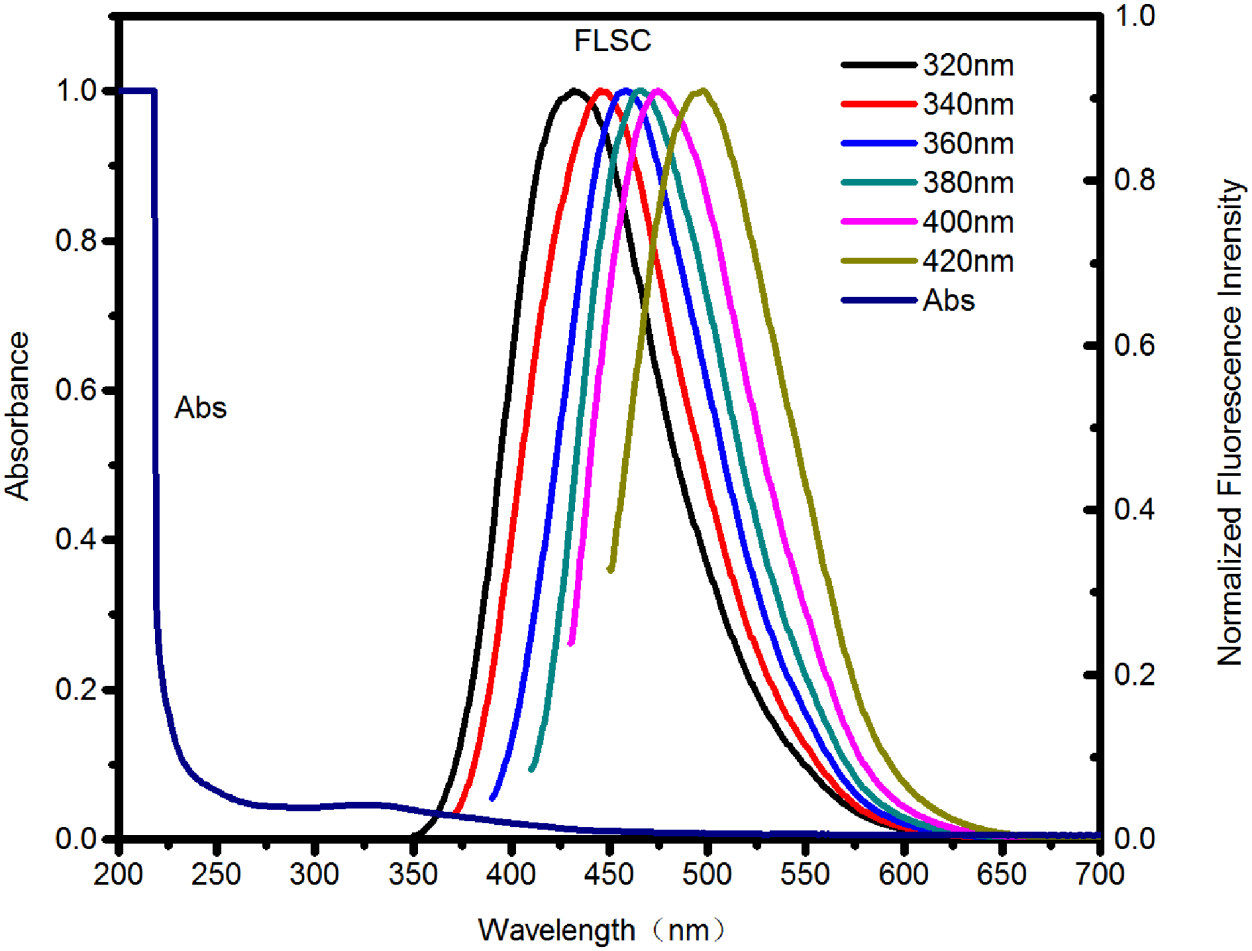
UV-Vis absorption spectra (Abs) and Fluorescence spectra (FLSC, excitation wavelengths as indicated) of the as-synthesized C-dots in aqueous solution.

As shown in Figure 2, an characteristic absorption band at 310–330 nm in the absorption spectrum of the carbon dots was attributed to n–π^*^ transition (Teng et al., 2014). The fluorescent spectra in Figure 2 exhibited an excitation-dependence feature (i.e., the emission was red-shifted with excitation wavelength increasing). The emission peak intensity firstly increased, and then decreased remarkably as excitation shifted to longer wavelength (see Figure S1). The optimum emission was revealed at 459 nm under excitation at 360 nm. Therefore, the emission of the carbon dots are controlled by surface states. And different emission centers on the surface result in excitation dependent emission (Chen et al., 2015; Dai et al., 2016; Kim et al., 2018). The fluorescent quantum yield of the carbon dots is 12.45% referencing to quinine sulfate (quantum yield 54.6%).

Photostability of the as-synthesized carbon dots was measured under continuous illumination at 360 nm with a 150 W Xe lamp in a fluorescence spectrophotometer. The fluorescence intensity exhibited negligible change within 50 min (Figure S2), suggesting that the as-synthesized carbon dots can keep photostable under light irradiation during detection and imaging.

**Figure 3:**
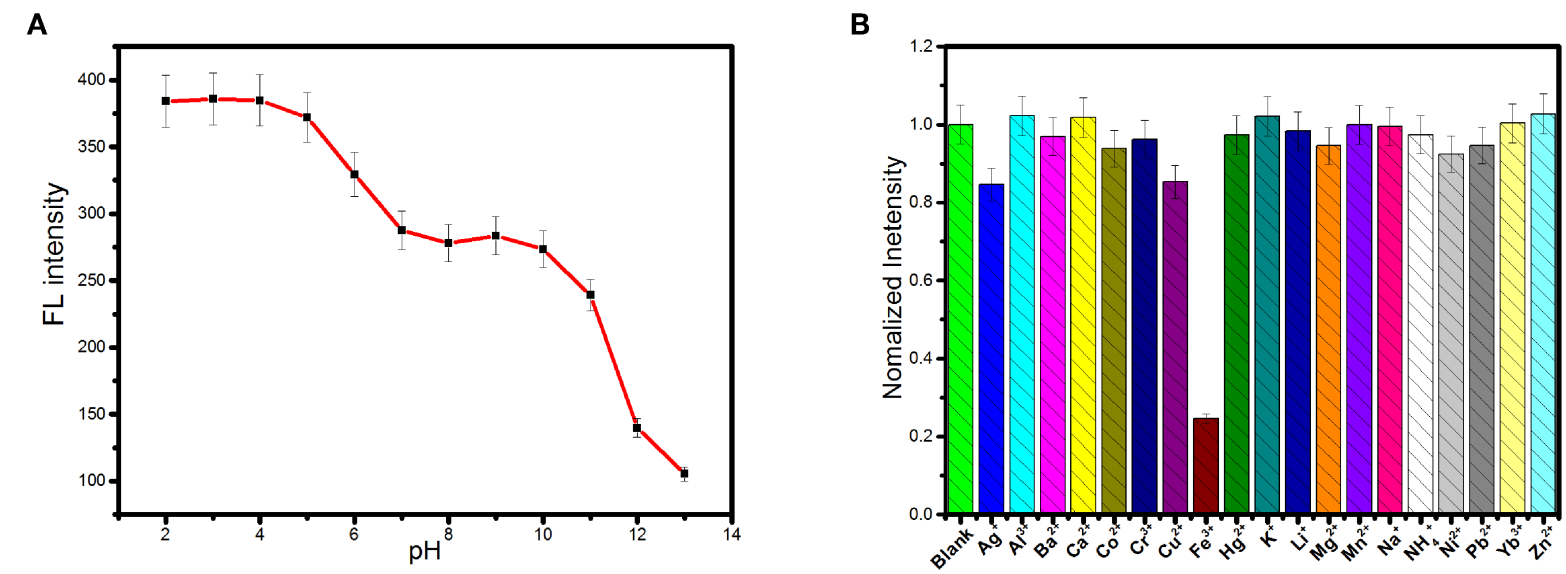
**(A)** The influence of pH on the fluorescence intensity of the as-synthesized carbon dots (λ_ex_ = 360 nm). **(B)** Fluorescence responses of 0.15 mg.mL^−1^ the as-synthesized carbon dots in Tris-HCl (0.1 M pH 7.0) upon the addition of various metal ions at the concentration of 0.5 mM.

The effect of pH on the fluorescence of the carbon dots was shown in Figure 3A. Fluorescence intensity decreased significantly as pH increased in the range of 5–7 and pH > 10. But fluorescence intensity of the carbon dots was independent on pH in the ranges of 2–4 and 7–9. Protonation–deprotonation occurring on the surface of the carbon dots with pH variation induced the changes in the surface charges, resulting in pH-dependence (Hu et al., 2014; Yuan et al., 2016; Liu et al., 2017). Therefore, if the carbon dots are used as bioimaging agents under the physiological pH condition, fluorescence intensity will hardly suffer from the pH variation.

Nineteen kinds of cations, including Li^+^, Na^+^, K^+^, ${\text{NH}}_{4}^{+}$, Al^3+^, Ag^+^, Ca^2+^, Fe^3+^, Ba^2+^, Co^2+^, Cr^3+^, Cu^2+^, Zn^2+^, Hg^2+^, Mg^2+^, Mn^2+^, Ni^2+^, Pb^2+^, and Yb^3+^, were selected as representatives to evaluate the effects of cations on the fluorescence of the carbon dots. As exhibited in Figure 3B, the fluorescence emission of the carbon dots could be significantly quenched by Fe^3+^ while the other cations exerted negligible effects. Therefore, the carbon dots respond to Fe^3+^ selectively in the system containing cations. In order to test the selectivity of the carbon dots to Fe^3+^ in complex environment, the other potential interferences from anions, biomolecules, and ionic strength were further tested. Figure S3 shows the results of the interferences of anions (Ac^−^, Br^−^, Cl^−^, ${\text{CO}}_{3}^{2-}$, F^−^, ${\text{HCO}}_{3}^{-}$, ${\text{NO}}_{3}^{-}$, ${\text{PO}}_{4}^{3-}$, and ${\text{SO}}_{4}^{2-}$) on fluorescence intensity of the carbon dots, from which no obvious changes were found in the presence of different anions by comparison with the blank. Furthermore, negligible influences of amino acids and glucose on fluorescence intensity of the carbon dots were found (see Figure S4). Additionally, an increase in ionic strength would not fluctuate the fluorescence of the carbon dots (Figure S5), suggesting that the detection of Fe^3+^ will not be affected by the variation of ionic strength. Overall, the carbon dots showed an excellent selectivity toward Fe^3+^ under complex condition.

The responsive time of the carbon dots toward Fe^3+^ ions was measured and the result is shown in Figure S6, which indicates that the response the carbon dots toward Fe^3+^ ions is balanced within 4 min.

The absorption spectra of the carbon dots in the presence of Fe^3+^ ions were collected in Figure S7A, which exhibits that absorbance at about 320 nm increases upon addition of Fe^3+^. Combined with the FT-IR results that there are hydroxyl, carboxyl, and amine groups on the surface of the as-synthesized carbon dots, it can infer that carbon dots can form stable complex with Fe^3+^ ions through carboxyl, resulting in fluorescence quenching due to photo-induced electron transfer and high selectivity of the carbon dots to Fe^3+^ (Chen et al., 2016; Li et al., 2017). The fluorescence lifetimes of the carbon dots in absence and presence Fe^3+^ ions were measured for the investigation of the mechanism of fluorescence quenching of Fe^3+^ ions toward carbon dots. As shown in Figure S7B, the fluorescence lifetime of the carbon dots ions nearly did not change upon addition of Fe^3+^, suggesting that the mechanism of fluorescence quenching of Fe^3+^ ions toward carbon dots can be attributed to static quenching (Jiang et al., 2019).

### Detection of Fe^3+^ in Aqueous Solution

**Figure 4:**
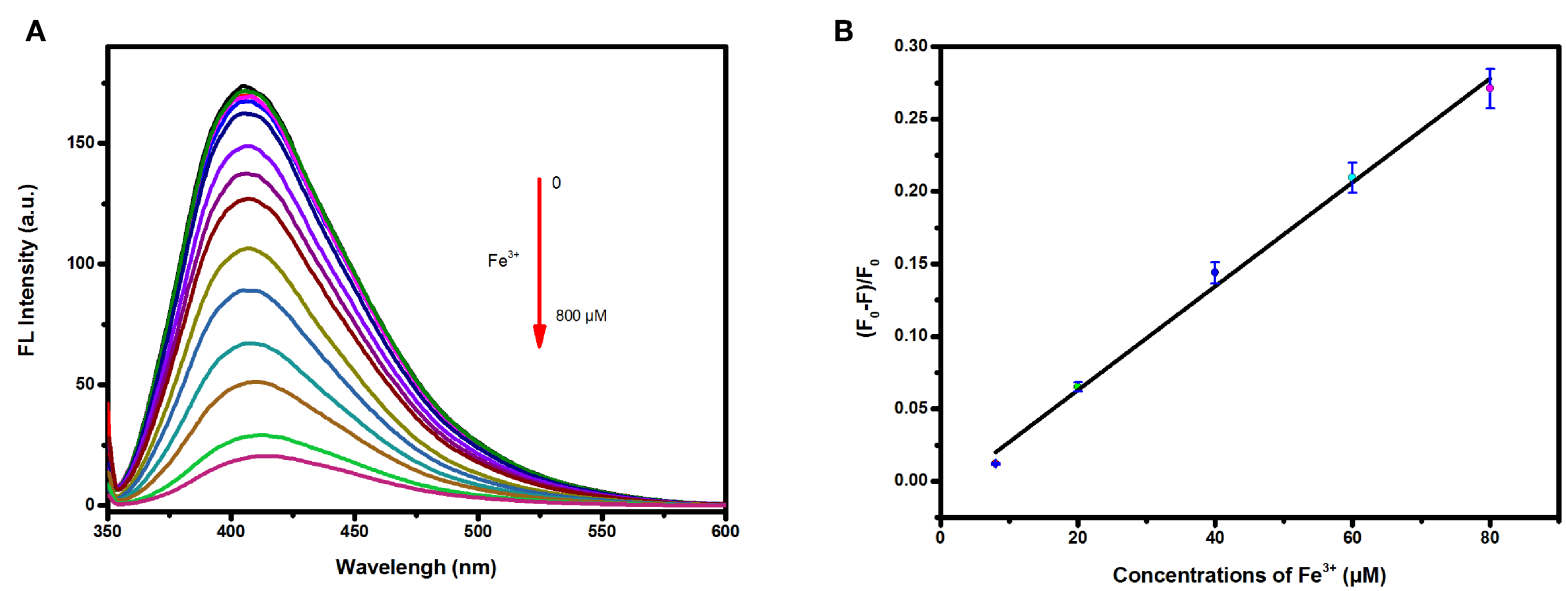
**(A)** Fluorescence response of the carbon dots in presence of tap water containing different concentrations of Fe^3+^ (from top to bottom: 0, 2, 4, 6, 8, 20, 40, 60, 80, 150, 200, 300, 400, 600, and 800 μM); **(B)** Corresponding relationship between (F_0_-F)/F_0_ and the concentrations of Fe^3+^ of tap water.

The fluorescence intensities of the carbon dots in Tris-HCl buffer solutions at pH = 7.0 containing Fe^3+^ at various concentrations were measured to study the detection sensitivity. As shown in Figure 4A, the emission of the carbon dots showed a decrease in intensity upon addition of Fe^3+^ with concentration increasing from 0 to 800 μM. A good linear relationship (Figure 4B) between (F_0_-F)/F_0_ and the concentration was found in the range of 8–80 μM (*R*^2^ = 0.9936). The linear equation was fitted as (F_0_-F)/F_0_ = 0.00358 c−0.00875 (c representing the concentration of Fe^3+^), and the limit of detection (LOD) was calculated to be 3.8 μM based on three times the standard deviation rule (LOD = 3Sd/s).

A comparison between the proposed method based on the carbon dots with the previously reported methods are listed in Table S1, which suggests that this method based on the carbon dots for the detections of Fe^3+^ is comparable or even better than those previously reported in literatures.

The carbon dots with high sensitivity and selectivity to Fe^3+^ might be directly applied for the detection of Fe^3+^ ions in real water samples. Therefore, quantitative detection of Fe^3+^ in tap water was conducted for evaluation the practical application of the carbon dots as a Fe^3+^ sensor. The tap water samples which were taken from our lab and spiked with Fe^3+^ at different concentrations without any pre-treatment. Then, the spiked Fe^3+^ was analyzed by the proposed method. As presented in Table 1, the recoveries of the spiked water samples are in the range from 90.6 to 107%, suggesting that the as-synthesized carbon dots can be employed as a promising sensor for Fe^3+^ in real water samples.

**Table 1:** Results for the detection of Fe^3+^ in tap water.

**Samples**	**Added Fe^3+^** **(μM)**	**Found Fe^3+^** **(μM)**	**Recovery (%)**	**RSD** **(*n* = 3, %)**
1	20	19.68 21.47 18.96	98.4 107 94.8	1.29
2	40	41.58 35.83 42.66	104 89.6 106	3.67
3	60	54.33 56.12 58.81	90.6 93.5 98.1	2.25

### Bioimaging of Fungal Cells and Intracellular Imaging of Fe^3+^ Inside Fungal Cells

**Figure 5:**
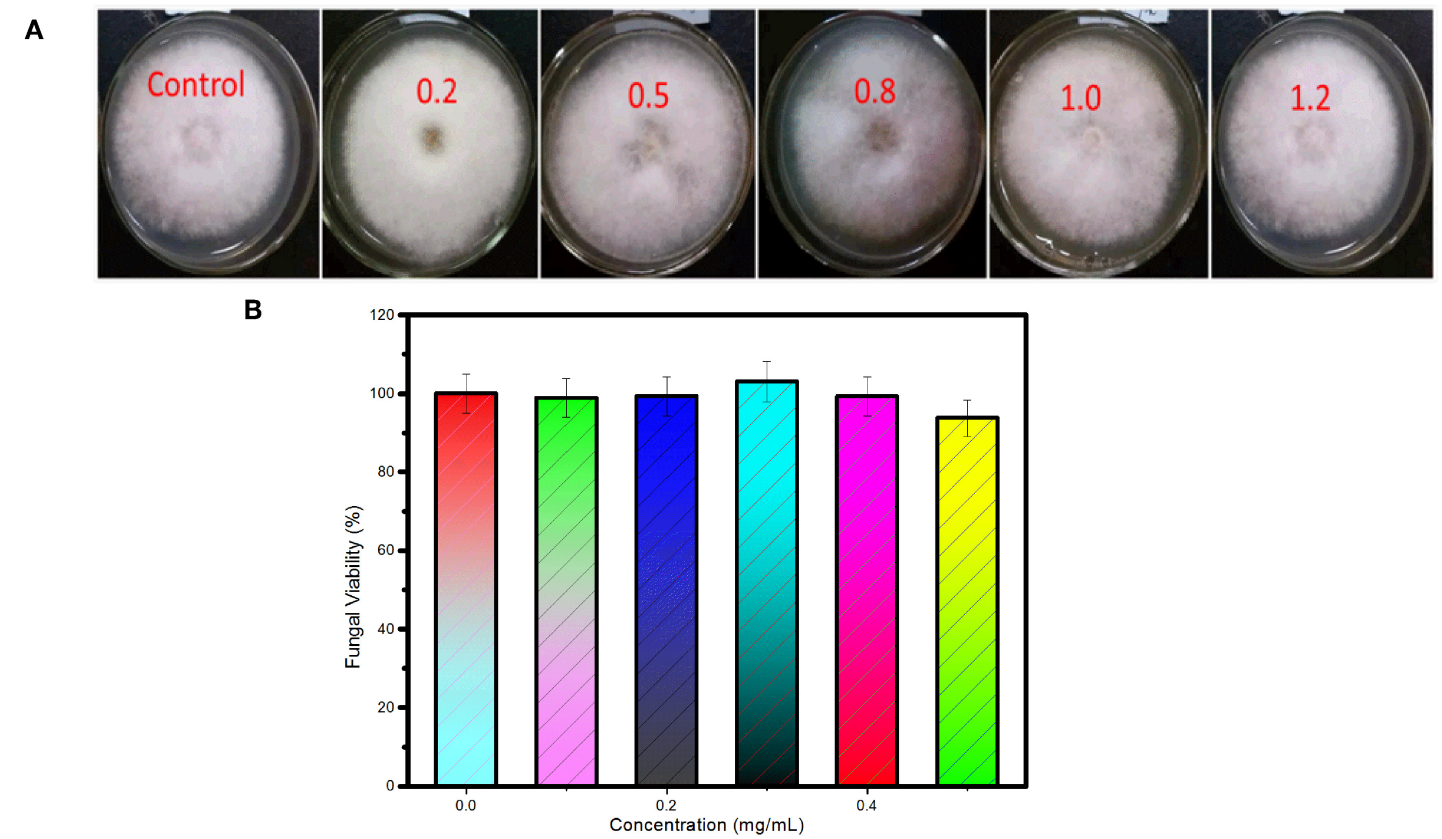
Cytotoxicity of the as-synthesized carbon dots to *C. gloeosporioides*. **(A)** Photos and **(B)** viability of the fungus growing on the medium containing the as-synthesized carbon dots at the concentrations of 0.2, 0.5, 0.8, 1.0, 1.2 mg/ml, respectively.

The cytotoxicity of the as-synthesized carbon dots to *C. gloeosporioides* fungus was tested by the method of growth rate. As shown in Figure 5A, fungal mycelia growing on the PDA media containing the as-synthesized carbon dots at the concentrations of 0.2, 0.5, 0.8, 1.0, 1.2 mg/mL, respectively, show no obvious difference compared with the control. The fungal viability is slightly reduced at the concentration of 1.2 mg/mL (Figure 5B). Therefore, the as-synthesized carbon dots are a kind of excellent biocompatible label for bioimaging of *C. gloeosporioides* fungus.

The potential of the as-synthesized carbon dots to be label agents for fluorescently imaging pathogenic fungal cells *(C. gloeosporioides)* on a confocal microscopy was explored. The isolated mycelia from liquid medium were incubated in the Tris-HCl buffer solutions in the presence of the as-synthesized carbon dots at different concentrations. After 36 h incubation at the room temperature, brightly illuminating fungal cells were observed by the confocal microscopy under excitation at 405 nm, which suggests that the carbon dots can readily enter the fungal cells and stain mycelia for imaging (see Figure S8). By analysis of the fluorescent intensity of the fluorescent images of the fungal cells, the internalization of the carbon dots into fungal cells exhibited dose dependence (Figure S8). The strongest fluorescent intensity of the fluorescent images was observed for the fungal cells incubated in the Tris-HCl buffer solution containing the as-synthesized carbon dots in the concentration range from 0.4 to 0.5 mg/mL. Thus, 0.4 mg/mL was chosen as the optimum concentration for subsequent cell labeling.

**Figure 6:**
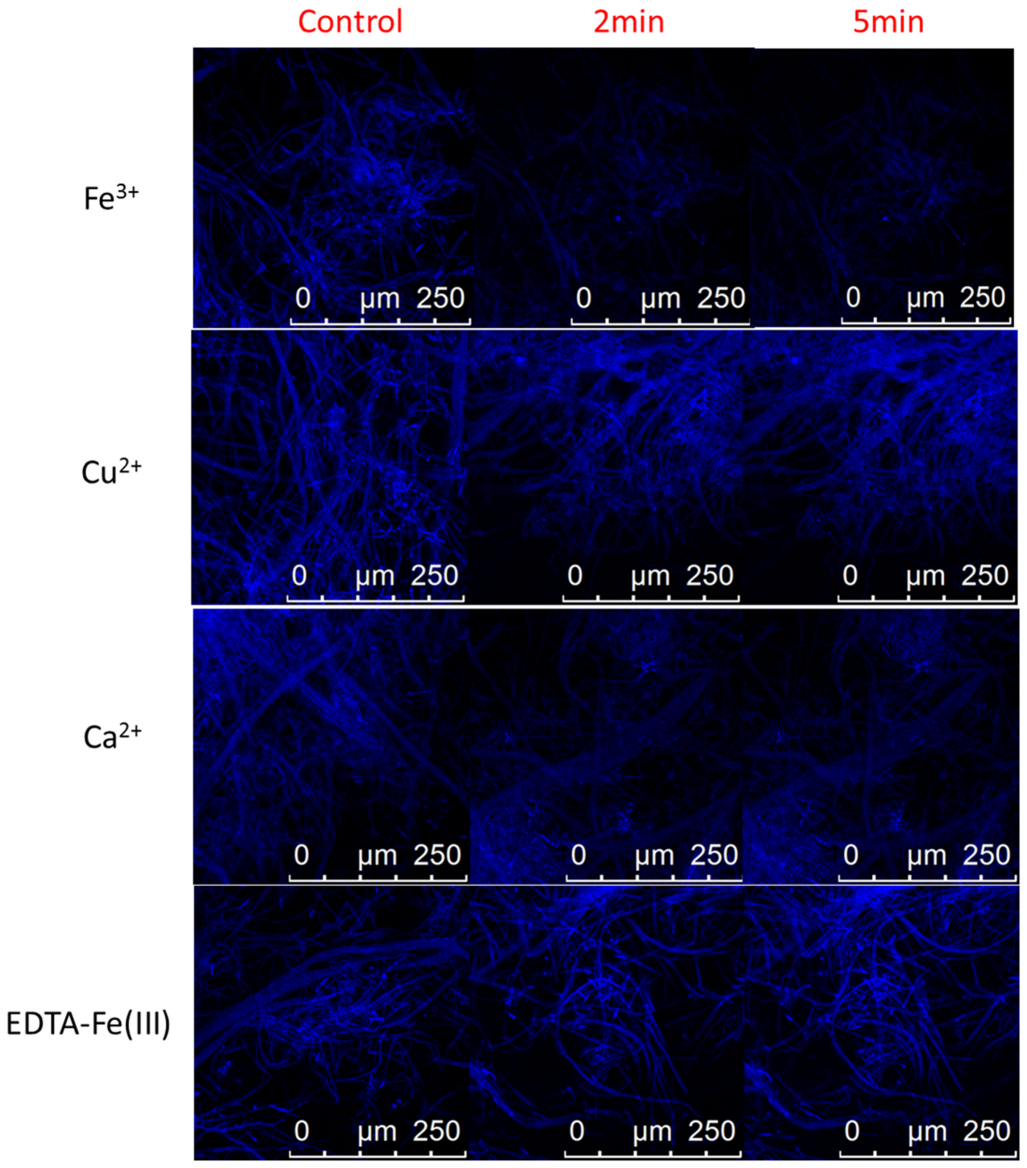
Representative confocal fluorescence images of plant pathogenic fungal cells (*C. gloeosporioides*) labeled with the carbon dots in Tris-HCl buffer solution (control) and Tris-HCl buffer solutions of 0.005 M Fe^3+^, Cu^2+^, Ca^2+^, and EDTA-Fe(III) complex.

In order to evaluate the feasibility of the as-synthesized carbon dots as fluorescent agent for detecting Fe^3+^ inside fungal cells, imaging was carried out on a confocal microscopy with the carbon dots labeled mycelia being incubated in Tris-HCl buffer solution containing 0.005 M Fe^3+^, and in Tris-HCl buffer solution containing 0.005 M EDTA-Fe(III) complex, Cu^2+^ and Ca^2+^ as comparison. As shown in Figure 6, the fungal cells incubated in blank Tris-HCl buffer solution showed strong intracellular fluorescence (control). But the intracellular fluorescence was significantly reduced in its intensity while the fungal cells were incubated in Tris-HCl buffer solution containing 0.005 M Fe^3+^ for 2 and 5 min. And incubation time had no obvious effect on the intracellular fluorescence intensity of the fungal cells (confocal photo taken at 5 min is not significantly different with that taken at 2 min), indicating that free Fe^3+^ can readily enter the fungal cells and effectively quench the fluorescence of the internalized carbon dots. Hence, the carbon dots show selective fluorescent response not only to free Fe^3+^ but also to the Fe(III) complex. However, the intracellular fluorescence intensity of the fungal cells incubated in Tris-HCl buffer solution containing the Fe(III) complex at a concentration of 0.005 M had not obvious change compared with that incubated in blank Tris-HCl buffer solution (Figure 6). Thus it can be speculated that EDTA may hinder Fe(III) to enter the fungal cells because iron uptake of fungal cells involves the use of iron siderophores secreted by fungi (David et al., 1992).

As discussed above, Cu^2+^ and Ca^2+^ have negligible effect on the fluorescence of the carbon dots in the Tris-HCl buffer solution. Herein, Cu^2+^ and Ca^2+^ were selected as the representative metal ions to evaluate the selectivity of the carbon dots for intracellular imaging of Fe^3+^ inside fungal cells. As presented in Figure 6, there was not obvious change in intracellular fluorescence intensity of the fungal cells incubated in Tris-HCl buffer solutions containing Cu^2+^ and Ca^2+^ compared with that in blank Tris-HCl buffer solution. Therefore, the other metal ions, such as Cu^2+^ and Ca^2+^, will not exert effect on the fluorescence of the internalized carbon dots in fungal cells during intracellular imaging of Fe^3+^. The as-synthesized carbon dots with high selectivity to Fe^3+^ have the potential to be developed as fluorescent probe for detection of Fe^3+^ inside fungal cells.

## Conclusion

The Fe^3+^-sensitive carbon dots can be synthesized by means of a microwave-assisted method using glutamic acid and ethylenediamine as reactants. The as-syntheszied carbon dots have the potential to be developed as a fluorescent chemosensor for detection of Fe^3+^ ions in aqueous solution because the dots showed selective response to Fe^3+^ ions in aqueous solution with a turn-off mode, and a good linear relationship was observed between (F_0_-F)/F_0_ and the Fe^3+^ concentrations of in the range of 8–80 μM. Furthermore, the carbon dots can act as fluorescent agent for fungal bioimaging since the fungal cells labeled by the carbon dots were brightly illuminated when imaged on the confocal microscopy with excitation by 405 nm laser pulses. In addition, the as-synthesized carbon dots can be explored as a probe with high selectivity for intracellular imaging of not only free Fe^3+^ but also Fe(III) complex inside fungal cells. Incubation in buffer solution with high concentration of Fe^3+^ will result in an increase in intracellular Fe^3+^ and fluorescence quenching of the carbon dots internalized in fungal cells, while extracellular incubation buffer solution of Fe(III)-EDTA complex will not exert obvious effect on the intracellular fluorescence intensity because EDTA might hinder Fe(III) to enter the fungal cells. In conclusion, the as-synthesized carbon dots can be a potential candidate for optical detection of Fe^3+^ in aqueous solution, bioimaging of fungal cells and intracellular imaging of Fe^3+^ inside fungal cells.

## Data Availability Statement

All datasets generated for this study are included in the article/Supplementary Material.

## Author Contributions

YC conceived and designed the experiments, performed the data analysis, and wrote the manuscript. XS played an important role in interpreting the results and contributed significantly to manuscript preparation. WP contributed reagents, materials, analysis tools. GY contributed to data analysis. JW contributed to the conception of the study, revised the manuscript, and approved the final version.

### Conflict of Interest

The authors declare that the research was conducted in the absence of any commercial or financial relationships that could be construed as a potential conflict of interest.
