# Supplementary material for: Fe3+-Sensitive Carbon Dots for Detection of Fe3+ in Aqueous Solution and Intracellular Imaging of Fe3+ Inside Fungal Cells
Source: Front Chem. 2020 Jan 15;7:911. doi: 10.3389/fchem.2019.00911 (PMC6974440; doi:10.3389/fchem.2019.00911)
Supplement: Supplementary file 1 [file Data_Sheet_1.pdf]

Supplementary Material

1 Materials

L-glutamic acid and anhydrous ethylenediamine were purchased from Lanji Technology Development Co, LTD (Shanghai, China). Quinine sulfate (98%, suitable for fluorescence) was obtained from Sinapharm Chemical Reagent Co. LTD (Shanghai, China). Ultrapure water was used throughout all the experiments purified through Water Purifier Nanopure water system (18.3MΩ.cm). All the other chemicals were of analytical grade and used without any further purification.

2 Characterization

UV-visible absorption spectra were obtained using a TU-1901 UV–Vis spectrophotometer (Pgeneral, China) at room temperature. Fluorescent measurements were performed with a fluorescence spectrometer F-2500 (Hitachi, Japan). The morphology and the microstructure of the CDs were analyzed by high-resolution transmission electron microscopy (HRTEM) on a JEOLJEM-2010 electron microscopy (JEOL, Japan) with an accelerating voltage of 200 kV. The carbon dots solutions were carefully dripped onto the 300-mesh copper grid coated with a lacy carbon film, and the solvent was evaporated at ambient temperature and pressure. The X-ray diffraction (XRD) of the carbon dots were recorded using a D8 ADVANCE X-ray diffractometer (Bruker AXS, German) with Cu-Kα radiation (40 kV, 40 mA, λ= 1.5418 Å) at a scanning rate of 1° min^{-1} in the range from 5° to 80°. The Fourier transform infrared (FTIR) spectrum was collected on a FT-IR200 spectrometer (Thermo, America) with the KBr pellet technique ranging from 400 to 4000 cm^{-1} at room temperature. The microscopic fluorescence images were acquired with a Leica TCS SP5 Laser Scanning Confocal Microscope(LSCM, Lieca, Germany) with the excitation wavelength of 405 nm. The quantum yields (QYs) of the prepared carbon dots were determined using quinine sulfate in 0.1 M H_2SO_4 (literature QY: 54.6%) as the standard sample by comparing the integrated fluorescence intensities (excitation at 360 nm) and absorbance values at 360 nm of the carbon dots aqueous solutions with those of quinine sulfate (Grabolle et al., 2009). The lifetime of carbon dots was acquired by FLS-1000 (Edinburgh Instruments).

3 Cytotoxicity of the carbon dots to the fungus

The cytotoxicity of the as-synthesized carbon dots to *C. gloeosporioides* fungus was assessed by the growth rate method. Mycelial disks (5 mm in diameter) of the test plant pathogenic fungus were placed in the center of sterile petri dishes (60 mm × 15 mm) which contained 10 mL potato dextrose agar (PDA) medium and the carbon dots at the concentrations of 0, 0.2, 0.5, 0.8, 1.0 and 1.2 mg/mL. The plates were incubated at 27 ± 2°C till the mycelia colonies of the control group reached the edge of the plates, and then mycelia growths with PDA containing the carbon dots were determined by measuring the colony diameter using decussating method. Each treatment was replicated 3 times. The survival percent (%) = A/A_0 ×100% was calculated to assess the fungal viability in the presence of the carbon dots, where A_0 was the mycelia growth diameter of the control group and A was the mycelia growth diameter of the carbon dots treated group.

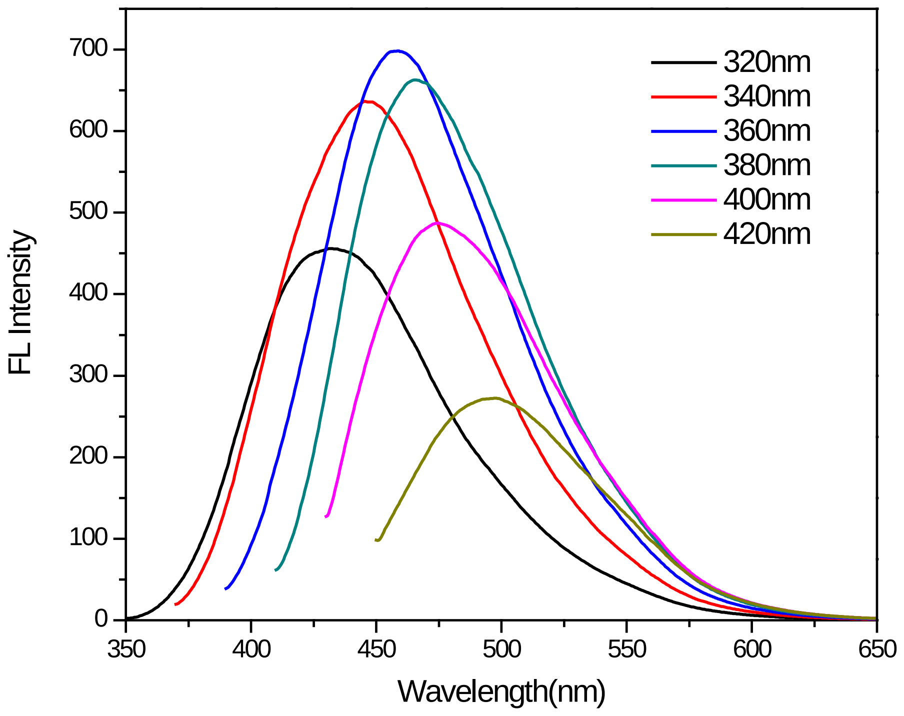

Fig. S1 Fluorescence spectra of the as-synthesized carbon dots in aqueous solution (excitation wavelengths as indicated).

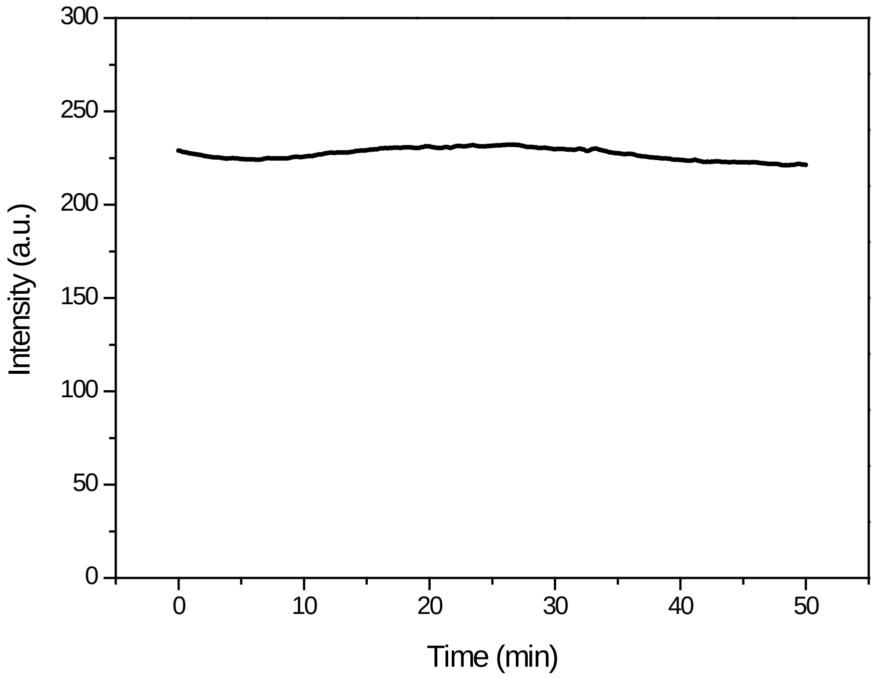

Fig. S2 Photostability test of the as-synthesized carbon dots in a fluorescence spectrophotometer with a 150 W Xe lamp under continuous excitation at 360 nm.

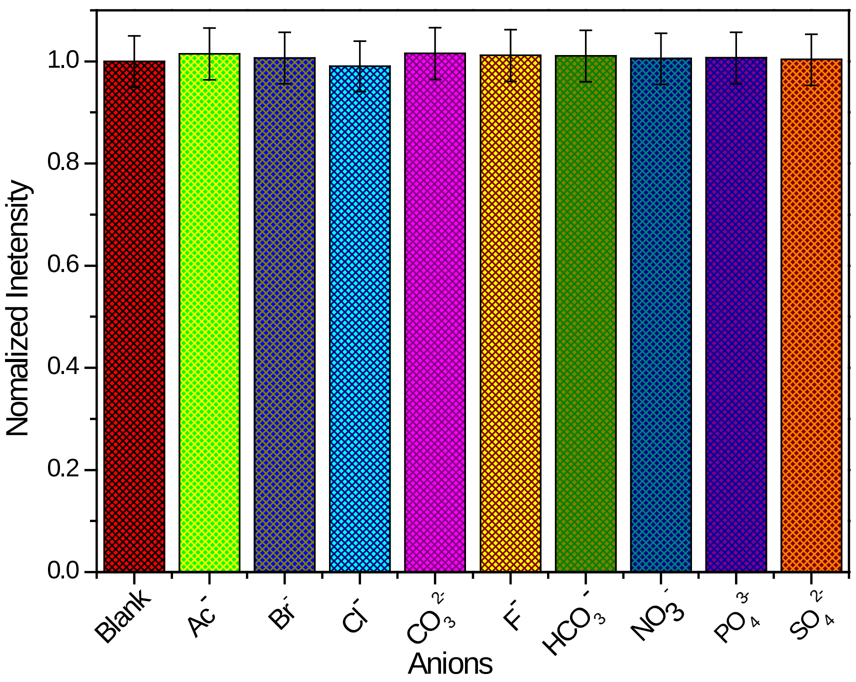

Fig. S3 Fluorescence responses of 0.15 mg mL^{-1} the as-synthesized carbon dots in Tris-HCl buffer solutions (0.1 M pH 7.0) upon the addition of various anions at the concentration of 0.5mM.

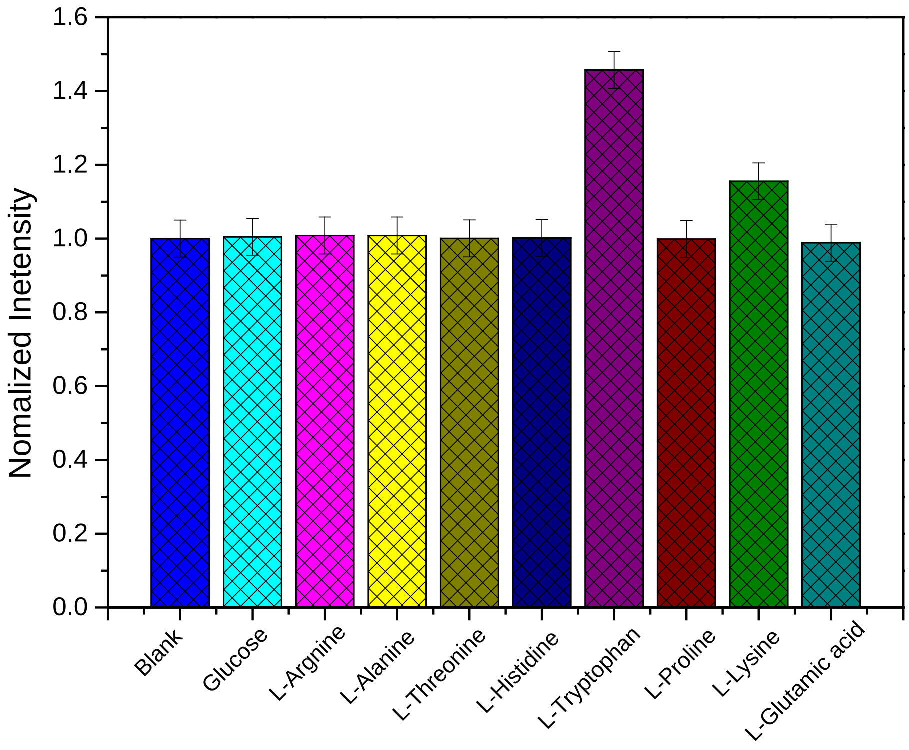

Fig. S4 Fluorescence responses of 0.15 mg mL^{-1} the as-synthesized carbon dots in the Tris-HCl buffer solutions (0.1M pH 7) upon addition of various biomolecules at the concentration of 0.5 mM.

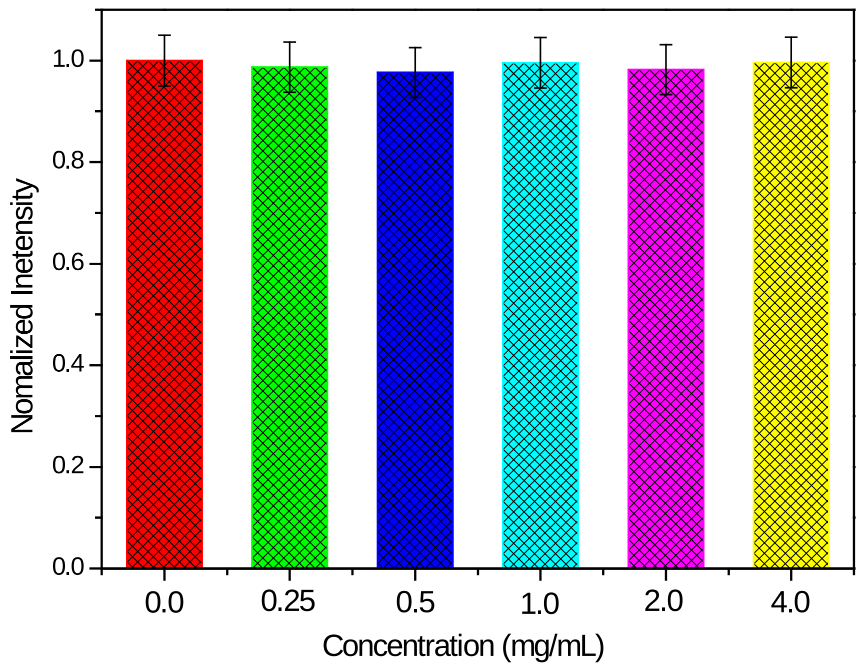

Fig. S5 Fluorescence intensity of 0.15 mg mL^{-1} the as-synthesized carbon dots in aqueous solution containing KCl with various ionic strengths (0, 0.25M, 0.5M, 1.0M, 2.0M, 4.0M).

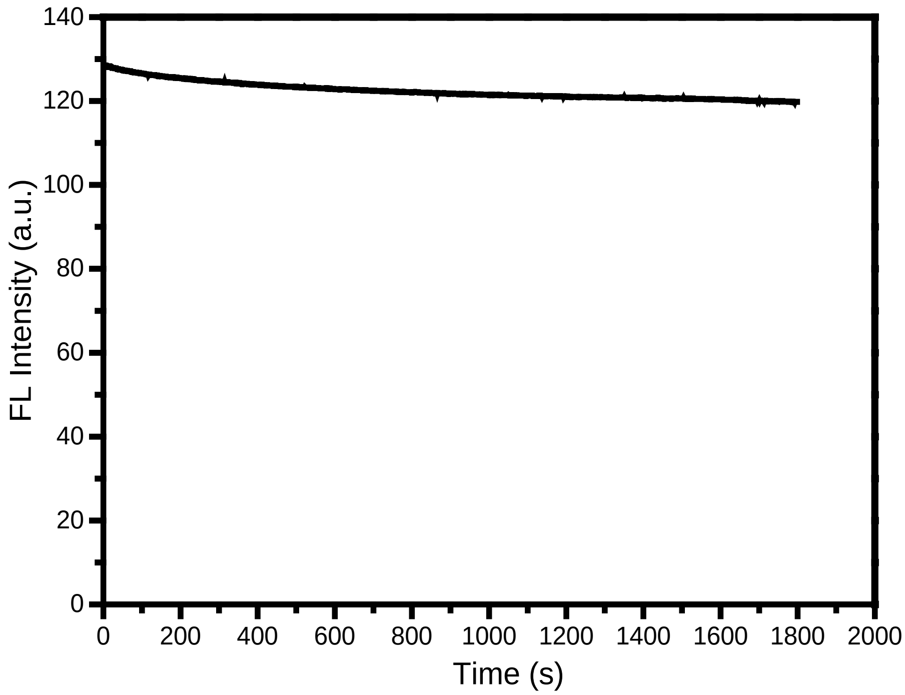

Fig. S6 The effect of time on the fluorescence intensity of the as-synthesized carbon dots after addition of Fe^{3+} under continuous excitation at 360 nm with a 150 W Xe lamp.

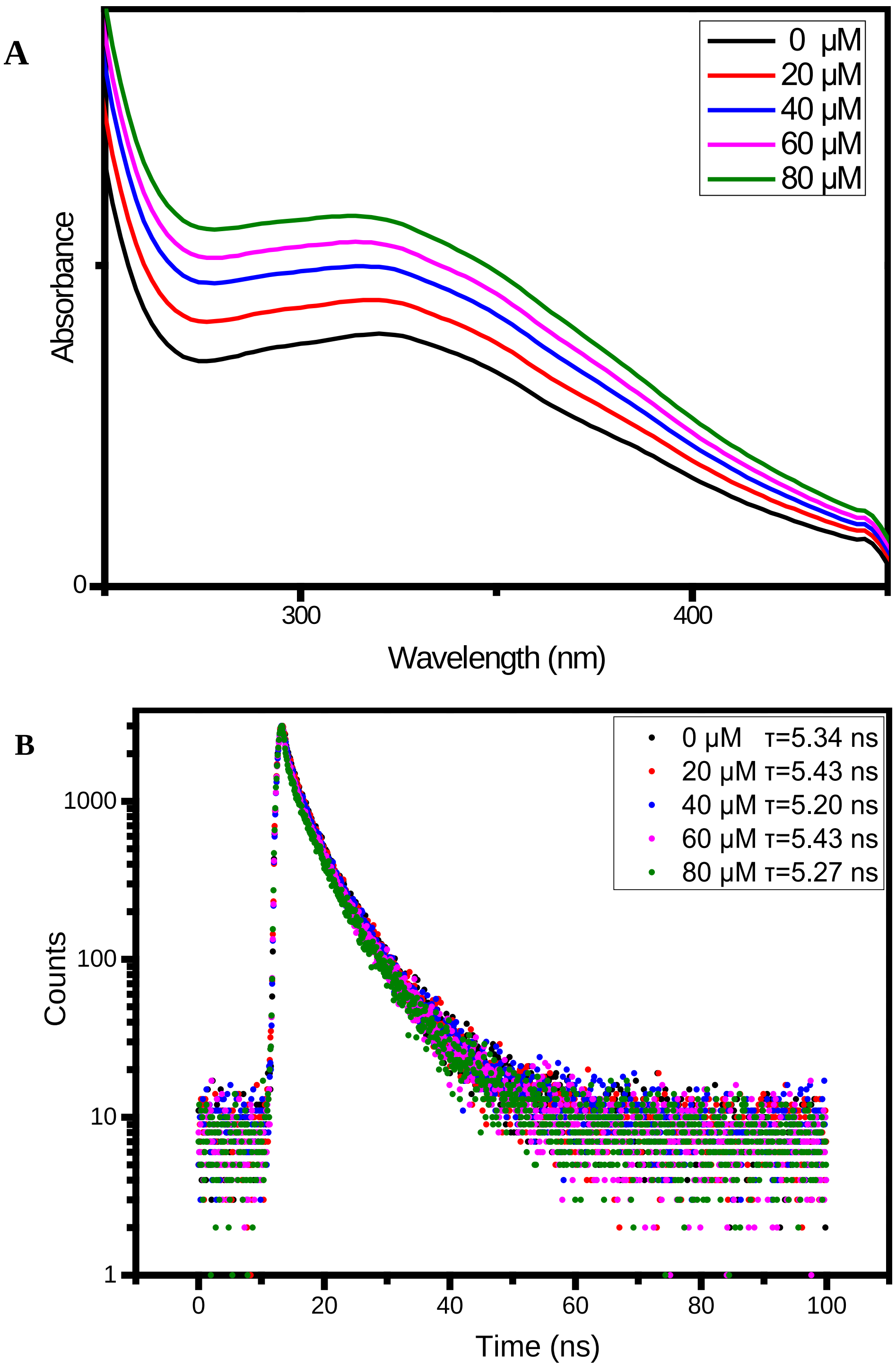

Fig. S7 (A) The absorption spectra of the carbon dots upon addition of Fe^{3+} (0, 20, 40, 60, 80 μM). (B) The fluorescence decay curves of the carbon dots in the Tris-HCl buffer solution containing 0, 20, 40, 60, 80 μM Fe^{3+}.

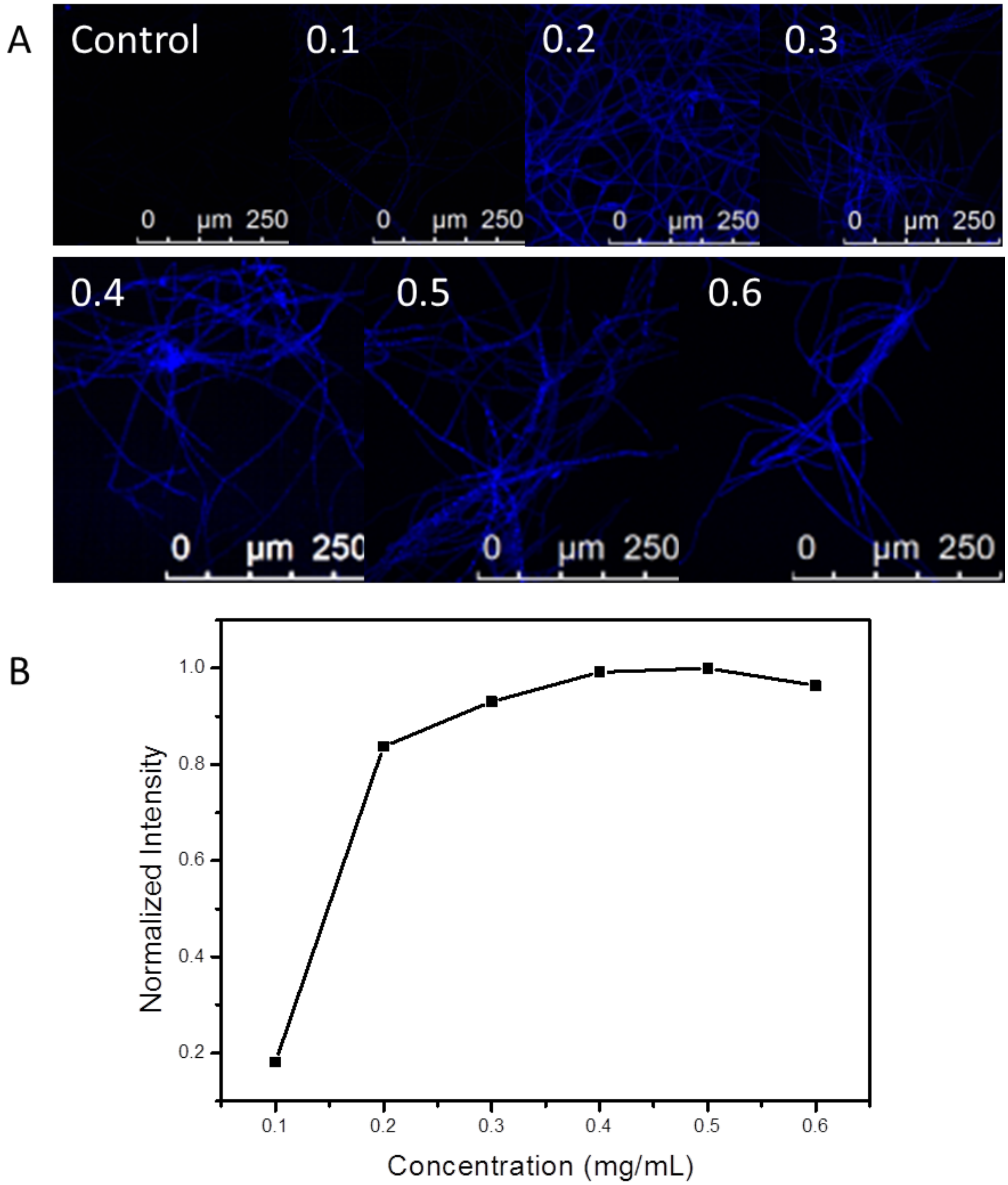

Fig. S8 (A) Confocal fluorescence images of plant pathogenic fungal cells (*C. gloeosporioides*) incubated in the Tris-HCl buffer solutions containing the as-synthesized carbon dots at different concentrations. (B) Quantitative analysis of the fluorescence intensity of the fungal cells.

Table S1 Comparison between the present method based on the as-prepared carbon dots with other methods for the detection of Fe^{3+}.

Method	Linear range (μM)	Limit of detection (μM)	References
Electrochemical	0.1-1.2	0.1	(Pang et al., 2019)
Chemiluminescence	<50	--	(Gomes et al., 2014)
UV-vis	30-1000	24.9	(Sadak et al., 2017)
GO nanosheets	14.32–143.2	17.9	(Wang et al., 2012)
Gold nanoclusters	5–1280	3.5	(Ho et al., 2012)
Copper nanoclusters	0.5–1000	0.34	(Feng et al., 2015)
Silver nanoclusters	0.5–20	0.12	(Chen et al., 2014)
GQDs	0–80	7.22	(Ananthanarayanan et al., 2014)
N-PNDs	0–30	0.1	(Lai et al., 2013)
F-CNPs	0–20	0.32	(Qu et al., 2013)
N,S-CDs	25–500	4	(Ding et al., 2014)
NCQDs	0-50	4.67	(Yu et al., 2016)
S-doped C-dots	0.1-500	0.1	(Xu et al., 2015)
Cu-CDs	0.001-200	0.001	(Xu et al., 2016)
CDs	8-80	3.8	This work

References:

Ananthanarayanan, A., Wang, X., Routh, P., Sana, B., Lim, S., Kim, D.H., et al. (2014). Facile Synthesis of Graphene Quantum Dots from 3D Graphene and their Application for Fe^{3+} Sensing. *Advanced Functional Materials* 24**,** 3021-3026. doi: 10.1002/adfm.201303441

Chen, Z., Lu, D., Zhang, G., Yang, J., Dong, C., and Shuang, S. (2014). Glutathione capped silver nanoclusters-based fluorescent probe for highly sensitive detection of Fe^{3+}. *Sensors and Actuators B: Chemical* 202**,** 631-637. doi: 10.1016/j.snb.2014.05.123

Ding, H., Wei, J.S., and Xiong, H.M. (2014). Nitrogen and sulfur co-doped carbon dots with strong blue luminescence. *Nanoscale* 6**,** 13817-13823. doi: 10.1039/c4nr04267k

Feng, J., Ju, Y., Liu, J., Zhang, H., and Chen, X. (2015). Polyethyleneimine-templated copper nanoclusters via ascorbic acid reduction approach as ferric ion sensor. *Anal Chim Acta* 854**,** 153-160. doi: 10.1016/j.aca.2014.11.024

Gomes, R., Roming, S., Przybilla, A., Meier, M.A.R., and Feldmann, C. (2014). Barium peroxide nanoparticles: synthesis, characterization and their use for actuating the luminol chemiluminescence. *Journal of Materials Chemistry C* 2, 1513-1518. doi: 10.1039/c3tc32273d

Grabolle, M., Spieles, M., Lesnyak, V., Gaponik, N., Eychmüller, A., and Resch-Genger, U. (2009). Determination of the Fluorescence Quantum Yield of Quantum Dots: Suitable Procedures and Achievable Uncertainties. *Analytical Chemistry* 81**,** 6285-6294. doi: 10.1021/ac900308v

Ho, J.A., Chang, H.C., and Su, W.T. (2012). DOPA-mediated reduction allows the facile synthesis of fluorescent gold nanoclusters for use as sensing probes for ferric ions. *Anal Chem* 84**,** 3246-3253. doi: 10.1021/ac203362g

Lai, T., Zheng, E., Chen, L., Wang, X., Kong, L., You, C., et al. (2013). Hybrid carbon source for producing nitrogen-doped polymer nanodots: one-pot hydrothermal synthesis, fluorescence enhancement and highly selective detection of Fe(iii). *Nanoscale* 5**,** 8015. doi: 10.1039/c3nr02014b

Pang, L.-Y., Wang, P., Gao, J.J., Wen, Y., and Liu, H. (2019). An active metal-organic anion framework with highly exposed SO_4^{2-} on {001} facets for the enhanced electrochemical detection of trace Fe^{3+}. *Journal of Electroanalytical Chemistry* 836**,** 85-93. doi: 10.1016/j.jelechem.2019.01.067

Qu, S., Chen, H., Zheng, X., Cao, J., and Liu, X. (2013). Ratiometric fluorescent nanosensor based on water soluble carbon nanodots with multiple sensing capacities. *Nanoscale* 5**,** 5514-5518. doi: 10.1039/c3nr00619k

Sadak, O., Sundramoorthy, A.K., and Gunasekaran, S. (2017). Highly selective colorimetric and electrochemical sensing of iron (III) using Nile red functionalized graphene film. *Biosens Bioelectron* 89**,** 430-436. doi: 10.1016/j.bios.2016.04.073

Wang, D., Wang, L., Dong, X., Shi, Z., and Jin, J. (2012). Chemically tailoring graphene oxides into fluorescent nanosheets for Fe^{3+} ion detection. *Carbon* 50**,** 2147-2154. doi: 10.1016/j.carbon.2012.01.021

Xu, Q., Pu, P., Zhao, J., Dong, C., Gao, C., Chen, Y., et al. (2015). Preparation of highly photoluminescent sulfur-doped carbon dots for Fe(iii) detection. *J. Mater. Chem*. A 3, 542-546. doi: 10.1039/c4ta05483k

Xu, Q., Wei, J., Wang, J., Liu, Y., Li, N., Chen, Y., et al. (2016). Facile synthesis of copper doped carbon dots and their application as a “turn-off” fluorescent probe in the detection of Fe^{3+} ions. *RSC Adv.* 6**,** 28745-28750. doi: 10.1039/c5ra27658f

Yu, J., Xu, C., Tian, Z., Lin, Y., and Shi, Z. (2016). Facilely synthesized N-doped carbon quantum dots with high fluorescent yield for sensing Fe^{3+}. *New J. Chem.* 40**,** 2083-2088. doi: 10.1039/c5nj03252k
